# Hypertensive Emergency in a Young Patient With Unilateral Adrenal Hyperplasia: A Case of Conn's Syndrome

**DOI:** 10.7759/cureus.17773

**Published:** 2021-09-06

**Authors:** Nagapratap Ganta, Suhrim Choe, Anish Kanukuntla, Priyaranjan Kata, Pramil Cheriyath

**Affiliations:** 1 Internal Medicine, Hackensack Meridian Ocean Medical Center, Brick, USA

**Keywords:** severe hypertension, hypokalemia, adrenal adenoma, plasma aldosterone concentration, unilateral adrenalectomy, hypertensive emergency, acth

## Abstract

Aldosterone is a mineralocorticoid hormone that maintains sodium and potassium homeostasis. Excess aldosterone secretion causes sodium influx and potassium outflow, leading to hypertension and in some cases hypokalemia. Conn's syndrome, or primary aldosteronism, is the most common cause of secondary hypertension, accounting for 20% or more of people with resistant hypertension. We present a young male with hypertension, blurry vision in the right eye, and hypokalemia who was on further investigation found to have an aldosterone-secreting adrenal adenoma. He was treated with retroperitoneoscopic right-sided adrenalectomy and his blood pressure improved. Conn's syndrome should be suspected in any hypertensive patient with hypokalemia. Adrenal venous sampling is the best investigation to measure aldosterone levels and also to lateralize the source. Surgical resection is the treatment of choice.

## Introduction

In 1954, Dr. Jerome W. Conn, a professor of Medicine at the University of Michigan, was asked to see a young patient with a seven-year history of muscle spasms, temporary paralysis, tetany, and weakness and a four-year history of hypertension with severe hypokalemia, mild hypernatremia, and alkalosis. He planned bilateral adrenalectomy based on his scientific philosophy on body acclimatization, suspecting an aldosterone-secreting tumor, after which the postoperative studies showed an almost total reversal of the preoperative metabolic and clinical abnormalities. At this point, Conn established for the first time the relationship among adrenal aldosterone-producing tumors, hypertension, and hypokalemia [1].

Aldosterone is a mineralocorticoid hormone that is important in maintaining sodium and potassium homeostasis. Excess secretion of aldosterone results in the influx of sodium and efflux of potassium resulting in hypertension and hypokalemia and some instances of hypomagnesemia [2]. Studies show that the incidence of primary aldosteronism among the population with essential hypertension is between 5% and 15%, and it could be around 10% [3]. Primary aldosteronism is the most common cause of secondary hypertension and has a prevalence of 20% among patients with resistant hypertension [4].

## Case presentation

A 31-year-old male presented to the emergency department with hypertension and blurry vision in the right eye suggestive of a hypertensive emergency requiring an intravenous infusion of nicardipine. One week earlier, he had presented with visual symptoms and was diagnosed with hemi retinal vein occlusion and optic nerve swelling. CT scan of the head revealed no signs of hemorrhage, and an MRI of the brain revealed no signs of a cerebrovascular accident. The patient’s medical history was significant for psoriasis and recently diagnosed hypertension. The patient required three medications to keep his blood pressure within an acceptable range at the time of his presentation.

**Figure 1:**
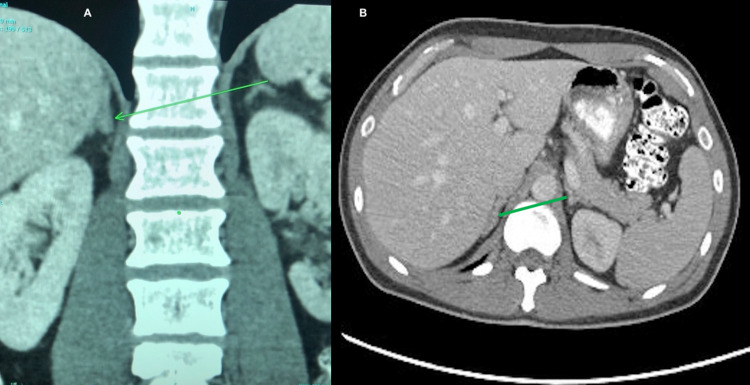
CT scan of the abdomen: (A) coronal section, (B) axial section showing asymmetric thickening of the medial lobe of the right adrenal gland

On his blood work, he was found to have hypokalemia, metabolic alkalosis, and high creatinine. A nephrologist and a primary medical team began the work-up for secondary hypertension after noticing signs of early-onset hypertension that required several antihypertensive drugs to control and blood work that revealed hypokalemia. The initial blood work showed elevated aldosterone, low renin, and high aldosterone-to-renin ratio. Further imaging studies with a CT scan of the abdomen showed asymmetric thickening of the medial lobe of the right adrenal gland (Figure 1).

Adrenal venous sampling was planned and scheduled for three weeks after the patient had stopped using spironolactone. Adrenal venous sampling done with adrenocorticotropic hormone stimulation showed right adrenal aldosterone/cortisol (A/C) ratio of 8.13:5.55, left adrenal A/C ratio of 0.29:0.26, and inferior vena cava (IVC) A/C ratio of 1.20:1. Because the left adrenal A/C ratio was lower than the IVC A/C ratio, it was established that the left adrenal gland was being suppressed contralaterally, resulting in the diagnosis of primary aldosteronism (Conn's syndrome) secondary to a right adrenal aldosterone-secreting adenoma.

A 0.8-cm aldosterone-secreting benign adrenocortical adenoma was found and confirmed by biopsy after the right adrenal gland was removed via retroperitoneoscopic adrenalectomy. The procedure was well tolerated by the patient, and there were no postoperative complications. Post adrenalectomy patient’s blood pressure was well controlled with a single medication as opposed to four before the surgery.

## Discussion

Complications of Conn’s syndrome are mainly due to sodium overload and fluid retention, which lead to chronic hypertension. Complications due to chronic hypertension are acute myocardial infarction, heart failure, end-organ damage like hypertensive retinopathy and/or renal damage, and stroke. Cardiovascular events like atrial fibrillation, non-fatal myocardial infarction, coronary artery disease, and heart failure are more common in patients with Conn’s syndrome on their first visit to clinic before the diagnosis. In addition to this, prolonged and high concentrations of aldosterone exposure on cardiovascular tissues can cause degenerative changes. Left ventricular hypertrophy is twice as common in patients with primary aldosteronism when compared to the general population. Aldosterone has proinflammatory, prothrombotic, and profibrotic qualities and these lead to end-organ damage. Overall combined effects of fluid retention, oxidative stress, inflammation, remodeling effects, hypertrophy, and fibrosis lead to arteriosclerosis, intimal thickening, plaque formation, and myocardial fibrosis [5]. Adrenalectomy is the treatment of choice for primary aldosteronism when it is clear that the aldosterone hypersecretion is lateralizing. However, postsurgical complications such as post-adrenalectomy syndrome and adrenal insufficiency are common, and due to its contribution to mortality and morbidity, the risk and benefit of adrenalectomy should be carefully considered for each individual before it is performed. Adrenal insufficiency can occur either with unilateral or bilateral adrenalectomy. When compared to open adrenalectomy, studies suggest that laparoscopic adrenalectomy has less morbidity and mortality [6].

Conn’s syndrome is suspected in hypertensive patients with hypokalemia. Primary hyperaldosteronism is a difficult condition to diagnose. Unilateral adrenal hyperplasia, as well as the presence of a contralateral large-size adrenal adenoma, are statistically uncommon. Surgical treatment is most likely required in young patients with adrenal adenomas (particularly large ones) and an abnormal hormonal profile. Blood work will reveal metabolic alkalosis with hypernatremia and hypokalemia. The significance of aldosterone-related cardiovascular complications should alert clinicians to increase the efforts in delivering early diagnosis and treatment. Case finding is a simple two-step process: at the first step, measure morning plasma aldosterone concentration and plasma renin activity. If the ratio is positive (>15) an aldosterone suppression test should be followed as a second step to confirm the diagnosis. The major advancement in treating unilateral adrenal adenoma is laparoscopic adrenalectomy. Many people may benefit from pharmacotherapy, especially with the advent of newer agents that are not anti-androgenic. Mineralocorticoid receptor antagonists are also available as a pharmacological treatment option [7].

## Conclusions

Primary hyperaldosteronism might pose a diagnostic challenge to physicians due to its wide range of signs and symptoms. Screening for hyperaldosteronism should be done more frequently in cases of young or resistant hypertension, with hypokalemia. The most accurate technique to determine renal potassium excretion is to collect a 24-hour urine sample. Renal response to hypokalemia would be a total potassium <15 mmol/day if a 24-hour urine sample was taken. The adrenal protocol should be followed while performing imaging studies to rule out massive tumors such as adrenocortical carcinomas. However, as imaging cannot distinguish between functioning and non-functioning adenomas, adrenal venous sampling is the best technique to lateralize the source of primary aldosteronism. Medical therapy is the recommended treatment for bilateral adrenal hyperplasia, but unilateral adrenalectomy is the preferred treatment for aldosterone-producing adenoma and unilateral adrenal hyperplasia.
